# Melanin biopolymer synthesis using a new melanogenic strain of *Flavobacterium kingsejongi* and a recombinant strain of *Escherichia coli* expressing 4-hydroxyphenylpyruvate dioxygenase from *F. kingsejongi*

**DOI:** 10.1186/s12934-022-01800-w

**Published:** 2022-05-02

**Authors:** Han Sae Lee, Jun Young Choi, Soon Jae Kwon, Eun Seo Park, Byeong M. Oh, Jong H. Kim, Pyung Cheon Lee

**Affiliations:** Department of Molecular Science and Technology and Department of Applied Chemistry and Biological Engineering, Ajou University, Woncheon-dong, Yeongtong-gu, Suwon, 16499 South Korea

**Keywords:** *Flavobacterium kingsejongi*, Melanin, 4-Hydroxyphenylpyruvate dioxygenase

## Abstract

**Background:**

Melanins are a heterologous group of biopolymeric pigments synthesized by diverse prokaryotes and eukaryotes and are widely utilized as bioactive materials and functional polymers in the biotechnology industry. Here, we report the high-level melanin production using a new melanogenic *Flavobacterium kingsejongi* strain and a recombinant *Escherichia coli* overexpressing *F. kingsejongi* 4-hydroxyphenylpyruvate dioxygenase (HPPD).

**Results:**

Melanin synthesis of *F. kingsejongi* strain was confirmed via melanin synthesis inhibition test, melanin solubility test, genome analysis, and structural analysis of purified melanin from both wild-type *F. kingsejongi* and recombinant *E. coli* expressing *F. kingsejongi* HPPD. The activity of *F. kingsejongi* HPPD was demonstrated via in vitro assays with 6 × His-tagged and native forms of HPPD. The specific activity of *F. kingsejongi* HPPD was 1.2 ± 0.03 μmol homogentisate/min/mg-protein. Bioreactor fermentation of *F. kingsejongi* produced a large amount of melanin with a titer of 6.07 ± 0.32 g/L, a conversion yield of 60% (0.6 ± 0.03 g melanin per gram tyrosine), and a productivity of 0.03 g/L·h, indicating its potential for industrial melanin production. Additionally, bioreactor fermentation of recombinant *E. coli* expressing *F. kingsejongi* HPPD produced melanin at a titer of 3.76 ± 0.30 g/L, a conversion yield of 38% (0.38 ± 0.03 g melanin per gram tyrosine), and a productivity of 0.04 g/L·h.

**Conclusions:**

Both strains showed sufficiently high fermentation capability to indicate their potential as platform strains for large-scale bacterial melanin production. Furthermore, *F. kingsejongi* strain could serve as a model to elucidate the regulation of melanin biosynthesis pathway and its networks with other cellular pathways, and to understand the cellular responses of melanin-producing bacteria to environmental changes, including nutrient starvation and other stresses.

**Supplementary Information:**

The online version contains supplementary material available at 10.1186/s12934-022-01800-w.

## Background

Natural biopolymers can be utilized to produce many valuable products, such as therapeutic drugs, cosmetics, food ingredients, biochemicals, and biofuels. In particular, bacterial biopolymers can be used as biomaterials for a wide range of applications [1]. Melanins are a group of heterologous polymeric pigments synthesized by diverse prokaryotes and eukaryotes [2]. In microbes, melanins have several beneficial roles, including protection against UV radiation [3], heavy metal toxicity [4], oxidative stress [5], and extreme temperatures [6].

**Fig. 1:**
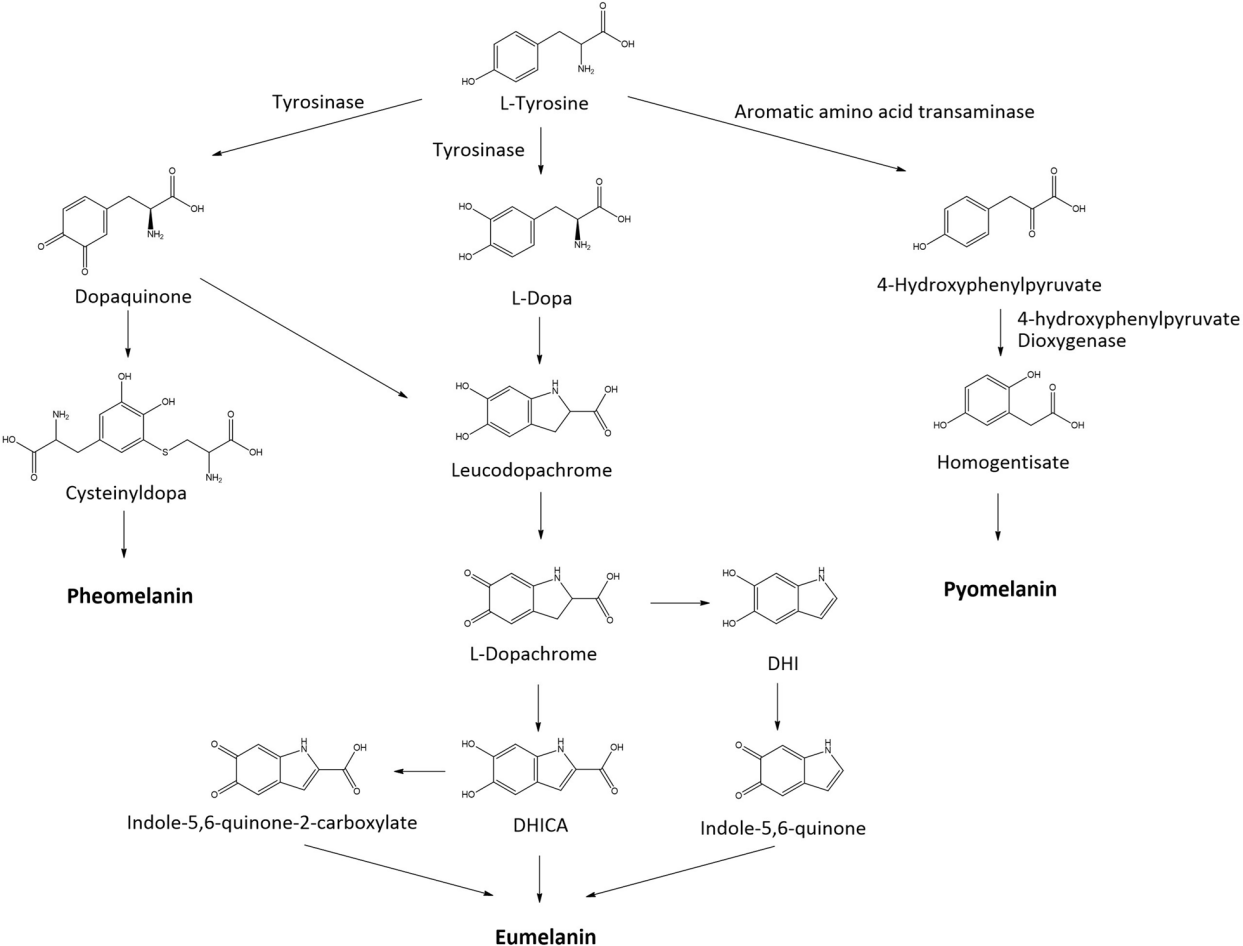
Biosynthesis of three classes of microbial melanin (eumelanin, pheomelanin, and pyomelanin) from the precursor l-tyrosine

Microbial melanins are heterologous, with four main types classified according to their chemical structures and biosynthetic pathways: eumelanin, pheomelanin, pyomelanin, and allomelanin [2]. Three of these types (eumelanin, pheomelanin, and pyomelanin) are biosynthesized from the same precursor, l-tyrosine, by sequential enzymatic actions (mainly through activity of tyrosinase, laccase, or 4-hydroxyphenylpyruvate dioxygenase [HPPD]) and autocatalysis (Fig. 1). Black-brown eumelanin (or DOPA-melanin) is synthesized from l-tyrosine, which is oxidatively transformed into L-3,4-dihydroxyphenylalanine (L-DOPA) by tyrosinase. L-DOPA is further oxidized to l-dopaquinone by tyrosinase. The resulting l-dopaquinone is then cyclized, oxidized, and polymerized to generate eumelanin [7]. Orange-yellow pheomelanin is also synthesized through oxidation of l-tyrosine to l-dopaquinone by tyrosinase. l-Dopaquinone is then cysteinylated into cysteinyl DOPA, which is further oxidized, cyclized, and polymerized to form pheomelanin [8]. The third class, pyomelanin, is synthesized from l-tyrosine via a different pathway, the homogentisate pathway, in which l-tyrosine is deaminated by tyrosine aminotransferase to form 4-hydroxyphenylpyruvate (4-HPP). 4-HPP is then further oxidized by HPPD to homogentisate, which is oxidized and polymerized to generate pyomelanin [9].

Microbial fermentation has been noted as a promising method for industrial synthesis of melanin because it is environmentally friendly and relatively easy to scale up. Melanin has a wide range of applications; it can be used in bioactive materials, such as antioxidants [10], sunscreen [11], and antimicrobial agents [12], and it can also be used in functional biomaterials, serving as a sensitizer in dye-sensitized solar cells [13] and as a scaffold polymer for metal nanoparticles [14]. In particular, pyomelanin has potential biotechnical applications such as bioremediation of metal-contaminated sites, extracellular electronic material in microbial fuel cells [15], and therapeutic agent for oxidative injury of neurocytes [16]. Therefore, systematic studies should be performed to assess the viability of microbial fermentation as a method for supplying melanin including pyomelanin for various industrial applications.

Pyomelanin is a black-brown pigment produced by diverse bacteria, fungi, and other organisms [17]. To date, studies on the production and physiological roles of pyomelanin have mainly focused on a few well-known wild-type microorganisms, such as *Ralstonia pickettii* [18], *Pseudomonas aeruginosa* [19–21], *Streptomyces avermitilis*[19–21], and *Aspergillus fumigatus* [19–21] and recombinant microorganisms expressing HPPD, such as *Escherichia coli* [18, 22]. Recently, a new *Flavobacterium kingsejongi* strain, isolated from Antarctic penguin feces [23], was found to produce a black-brown pigment. To the best of our knowledge, there have been no previous reports on the production and biosynthetic regulation of melanin in *Flavobacterium* strains.

In this study, we evaluated a new melanogenic *F. kingsejongi* strain and a recombinant *Escherichia coli* strain expressing *F. kingsejongi* HPPD, aiming to understand the melanin biosynthesis pathway in *F. kingsejongi* and its regulation and to evaluate the two strains as feasible hosts for melanin production in a bioreactor.

## Results and discussion

### Characteristics of the brownish-black pigment from *F. kingsejongi*

*Flavobacterium kingsejongi* produced a brownish-black pigment both on LB agar plates and in LB culture medium. The pigment was preliminarily characterized as a melanin using previously reported characterization methods [2]. The solubility of the partially purified pigment from *F. kingsejongi* was very similar to that of commercially available melanin (Additional file 1: Table S1), suggesting that it belonged to one of the melanin classes. Next, to verify our hypothesis that *F. kingsejongi* had a melanin biosynthesis pathway, we monitored the pigmentation of *F. kingsejongi* grown on LB agar plates supplemented with: (1) 1 g/L of tyrosine (known as an enhancer and precursor of melanin biosynthesis) [17], (2) 100 mg/L of kojic acid (known as an inhibitor of melanin biosynthesis) [24], and (3) both tyrosine (1 g/L) and kojic acid (100 mg/L). Similar to melanin-producing *S. avermitilis* (the positive control), *F. kingsejongi* showed markedly enhanced pigmentation intensity on the tyrosine/LB agar plate, but severely reduced pigmentation on both kojic acid/LB agar plates and tyrosine/kojic acid/LB agar plates. *E. coli* grown on these three types of plates (negative control) did not show pigmentation (Additional file 1: Fig. S1). Thus, *F. kingsejongi* was confirmed to have a melanin biosynthesis pathway.

### Chemical characterization of the brownish-black pigment from *F. kingsejongi*

**Fig. 2:**
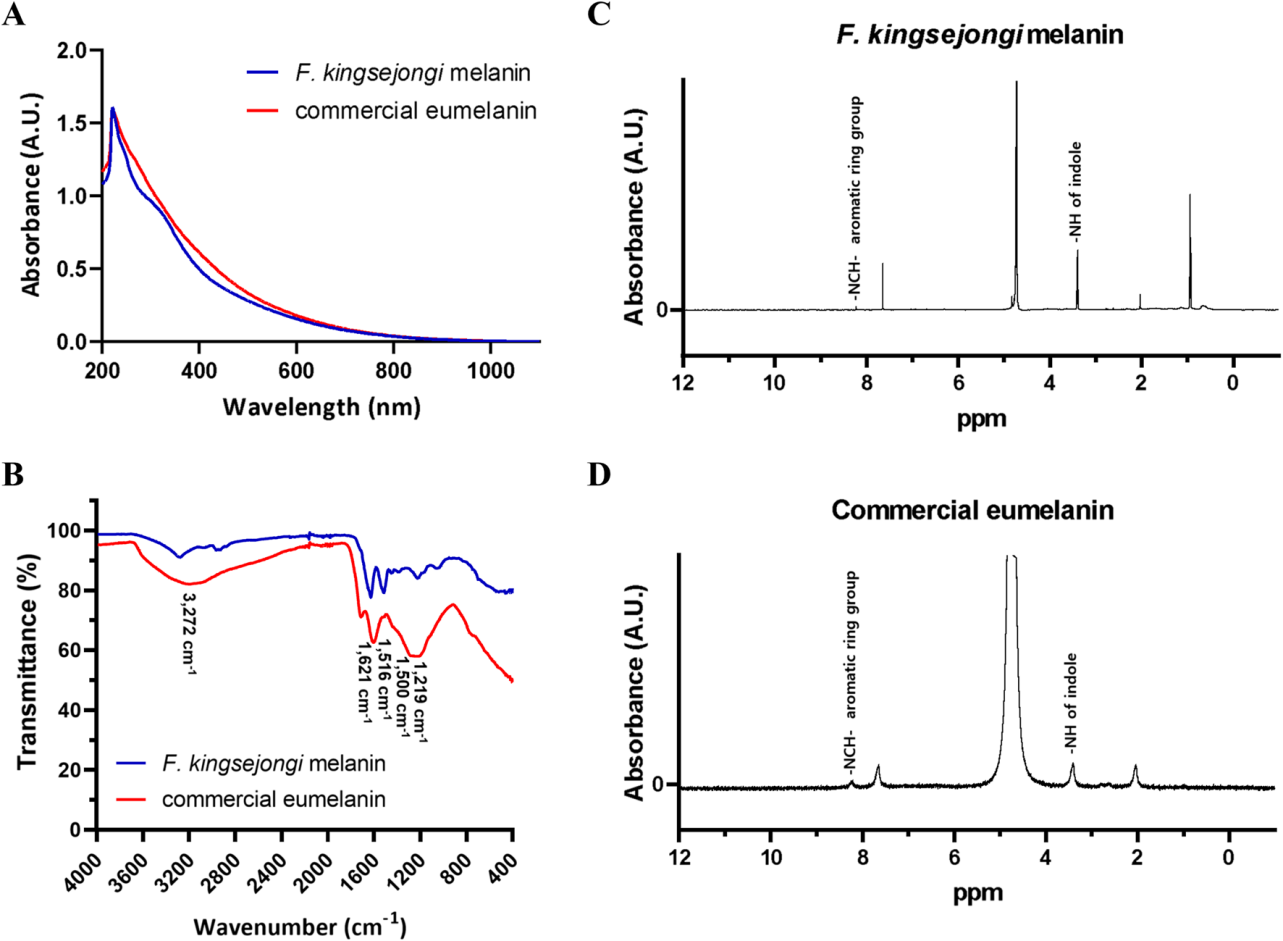
Structural analysis of the melanin purified from the *F. kingsejongi* culture broth. **A** UV–vis spectra analysis of *F. kingsejongi* melanin and commercial eumelanin (as a control). **B** FT-IR spectra of *F. kingsejongi* melanin and commercial eumelanin (as a control). **C** ^1^H NMR spectra of *F. kingsejongi* melanin. **D** ^1^H NMR spectra of commercial melanin (as a control)

As the brownish-black pigment produced by *F. kingsejongi* was thought to be a melanin, the chemical properties of the purified pigment were characterized by comparison with a commercially available eumelanin as standard. The commercial eumelanin and the *F. kingsejongi* pigment exhibited highly similar broad-band UV–vis spectra with an *l*_max_ at 221 nm (Fig. 2A). This unique *l*_max_ at 221 nm corresponds well with the general properties of melanin structures [25]. FT-IR spectroscopy analysis further revealed that the functional groups characterizing melanins were present in the *F. kingsejongi* pigment (Fig. 2B). First, there was a characteristic broad band around 3272 cm^−1^, corresponding to stretching vibrations of –OH and –NH groups; secondly, there were resolvable peaks at 1707, 1621, and 1516 cm^−1^, corresponding to aromatic rings; thirdly, there were peaks at 1500–1350 cm^−1^ caused by the bending vibration of the –NH group and the stretching vibration of the C–N group in an indole unit; and fourthly, the stretching vibration of a phenolic –OH group was detected at 1219 cm^−1^. Compared to the commercial eumelanin, the *F. kingsejongi* melanin showed characteristic peaks at 2967–2874 cm^−1^, 1081–1040 cm^−1^, and 873 cm^−1^, which can be attributed to asymmetrical stretching vibration of CH_2_, symmetric contraction vibration of C–O–C, and out-of-plane bending vibration of –CH [25–27]. Finally, ^1^H NMR spectra analysis of the *F. kingsejongi* melanin (Fig. 2C) showed a broad peak at ~ 7.0 ppm, corresponding to the aromatic protons of indole and pyrrole units, and a sharp peak at 8.4 ppm, corresponding to the protons of an –NCH– aromatic ring group [27, 28]. The characteristic sharp peak at 8.4 ppm was also present in the ^1^H NMR spectrum of the melanin standard (Fig. 2D).

### Identification and heterologous expression of the putative eumelanin-synthesizing gene of *F. kingsejongi*

**Fig. 3:**
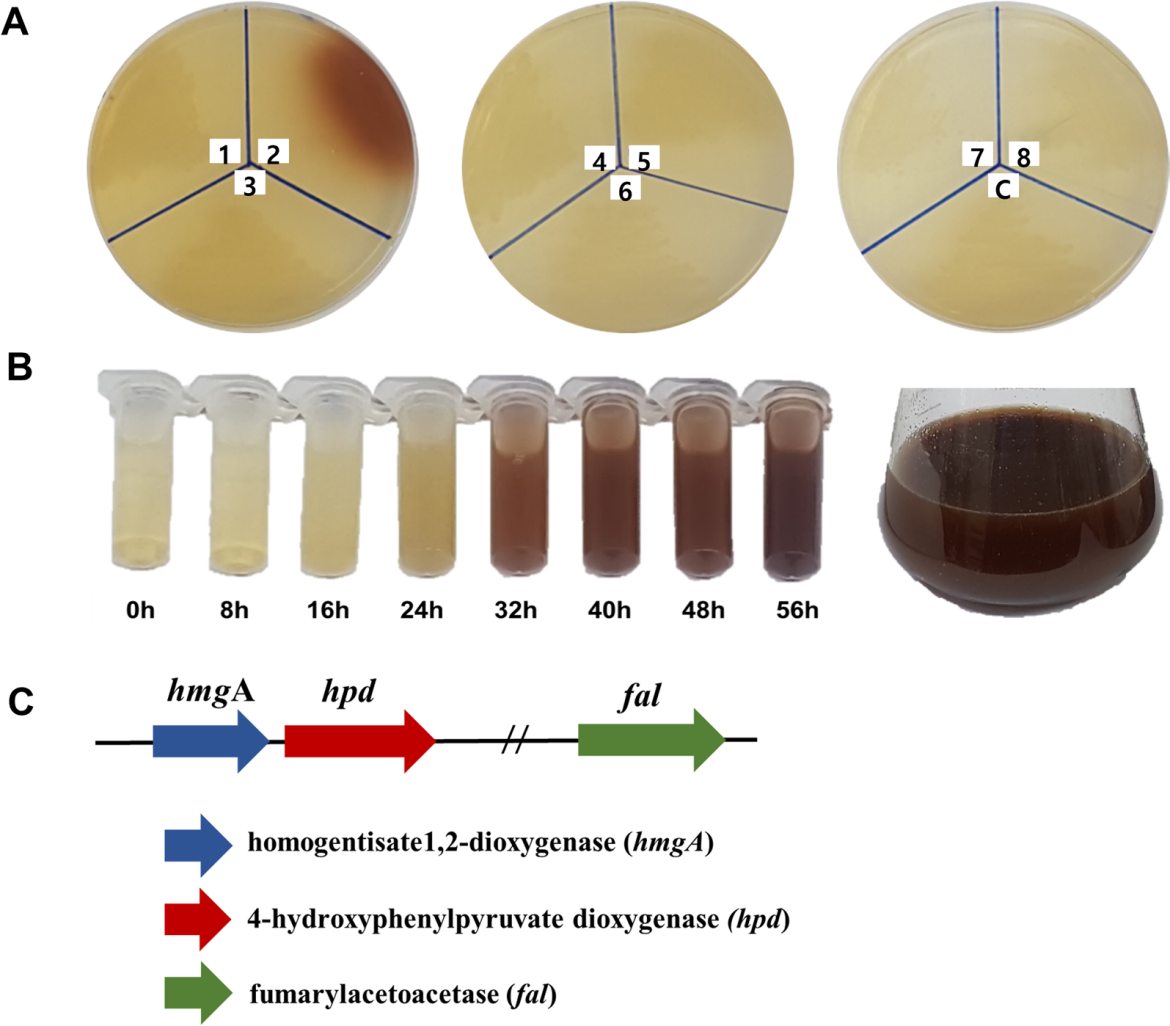
Heterologous expression of the eight putative proteins from *F. kingsejongi* involved in melanin synthesis in *E. coli*; organization of genes involving homogentisate pathway of *F. kingsejongi*. **A** *E. coli* cultures expressing eight *F. kingsejongi* candidate genes after 2 days of growth at 30 °C on LB agar plates supplemented with 1 g/L tyrosine. Plate sections are designated as follows: 1, tryptophan 2,3-dioxygenase; 2, 4-hydroxyphenylpyruvate dioxygenase (HPPD); 3, homogentisate 1,2-dioxygenase; 4, 3-hydroxyanthranilate 3,4-dioxygenase; 5, phytanoyl-CoA dioxygenase; 6, phytanoyl-CoA dioxygenase; 7, putative dioxygenase; 8, hypothetical protein; C, empty plasmid (negative control). **B** Time-course monitoring of culture broth of *E. coli* expressing the putative HPPD. **C** Organization of genes encoding homogentisate pathway enzymes in *F. kingsejongi*

Having confirmed that *F. kingsejongi* produces melanin, we deemed its melanin biosynthesis pathway (Fig. 1) worthy of further characterization for the purposes of molecular studies and metabolic engineering. Melanins are biosynthesized via two main routes involving tyrosinase (i.e., monophenol monooxygenase) and dioxygenase, and thus, the amino acid sequence of *S. lincolnensis* tyrosinase was used as a query sequence to search for a putative tyrosinase in the genome of *F. kingsejongi* [29]. Unfortunately, no tyrosinase candidate was detected. Therefore, the search range was extended to putative dioxygenases responsible for melanin synthesis. Eight candidate genes possibly involved in melanin biosynthesis were selected: genes encoding tryptophan 2,3-dioxygenase, HPPD, homogentisate 1,2-dioxygenase, 3-hydroxyanthranilate 3,4-dioxygenase, phytanoyl-CoA dioxygenase, phytanoyl-CoA dioxygenase, a putative dioxygenase, and a hypothetical protein. An experiment was performed using recombinant *E. coli* strains constitutively expressing each of the eight genes and an empty plasmid (as a control); only the *E. coli* strain expressing putative HPPD formed brown-colored colonies on LB agar plates supplemented with 1 g/L tyrosine and maintained at 30 °C (Fig. 3A). In LB broth supplemented with 1 g/L tyrosine, the *E. coli* strain expressing putative HPPD secreted brown pigments, turning the culture broth dark brown after 24 h (Fig. 3B). UV–vis, FT-IR spectroscopy, and ^1^H NMR spectroscopy revealed that the brown pigments from the *E. coli* strain expressing putative HPPD were structurally very similar to the melanin from *F. kingsejongi* (Additional file 1: Fig. S2). Furthermore, the pigmentation of both *F. kingsejongi* and the recombinant *E. coli* expressing HPPD were severely reduced in medium supplemented with the well-known HPPD inhibitor sulcotrione (Additional file 1: Fig. S3), proving that HPPD was responsible for pyomelanin biosynthesis in the non-melanogenic *E. coli* [22, 30]. These results strongly indicate that the putative HPPD catalyzes pyomelanin biosynthesis from tyrosine in *F. kingsejongi.* As pyomelanin is produced by HPPD activity in the homogentisate pathway [31], genome mining [32] was performed to identify genes involved in the homogentisate pathway in the genome of *F. kingsejongi*. Genome mining revealed that the putative single-copy gene encoding HPPD was present in the genome of *F. kingsejongi*, together with two genes encoding other homogentisate pathway enzymes (homogentisate 1,2-dioxygenase and fumarylacetoacetase) (Fig. 3C).

### In vitro activity of purified 6 × His-tagged HPPD and native HPPD in crude protein extract

**Fig. 4:**
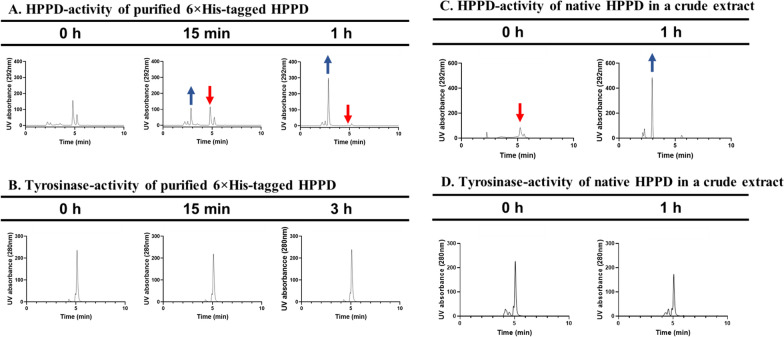
In vitro activity of purified 6 × His-tagged HPPD and native HPPD in crude protein extract. **A** HPPD activity was investigated using 4-HPP as a substrate for purified 6 × His-tagged HPPD. Red and blue arrows indicate peaks corresponding to homogentisate (product) and 4-HPP (substrate), respectively. **B** Tyrosinase activity was investigated using tyrosine as a substrate for purified 6 × His-tagged HPPD. **C** HPPD activity was investigated using 4-HPP as a substrate for native HPPD in a crude protein extract. Red and blue arrows indicate peaks corresponding to homogentisate (product) and 4-HPP (substrate), respectively. **D** Tyrosinase activity was investigated using tyrosine as a substrate for native HPPD in a crude protein extract

**Fig. 5:**
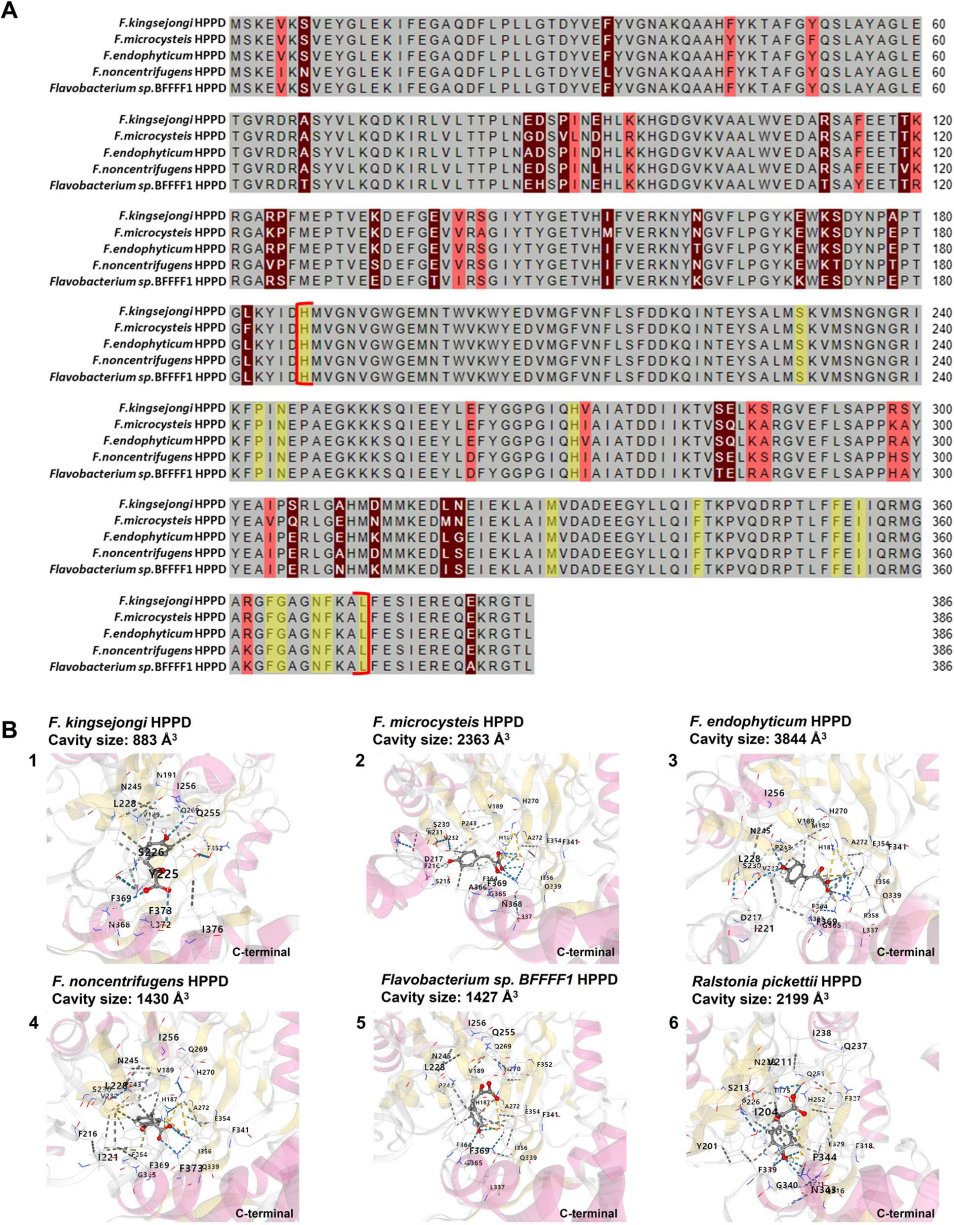
Multialignment of amino acid sequences of five *Flavobacterium* HPPD and homology modelling of the five *Flavobacterium* HPPDs and *R. pickettii* HPPD. **A** Amino acid sequences of *F. kingsejongi* HPPD and the four phylogenically close *Flavobacterium* HPPDs were aligned. The grey background represents 100% homology identities of amino acids between the five HPPDs, the red background represents 80% homology identities, and the brown background represents 50% homology identities. Amino acid sequences braced in red brackets represent the active site for 4-HPP binding. The yellow background indicates the binding residues in the active site for HPPD-4-HPP complex. **B** 3D molecular docking models for the HPPD-4-HPP complexes in the active site region of the HPPDs of *F. kingsejongi* (1), *F. microcysteis* (2), *F. endophyticum* (3), *F. noncentrifugens* (4), *Flavobacterium* sp. BFFFF1 (5), and *R. pickettii* (6) are presented. A molecular atom stick model of the substrate 4-HPP is presented. Hydrophobic, hydrogen bonding, and ionic interactions are indicated as dashed grey, blue, and yellow lines, respectively. Cavity sizes of each active site of the protein–ligand models are presented

To confirm that the observed in vivo activity was due to the putative HPPD, an in vitro assay of HPPD activity was performed with both purified 6 × His-tagged HPPD (Additional file 1: Fig. S4) and native HPPD in a crude protein extract of *E. coli*. Tyrosine and 4-HPP were used as substrates for determining tyrosinase-like activity and HPPD-like activity, respectively. Thus, there were four reaction conditions: (1) 6 × His-tagged HPPD + 4-HPP, (2) 6 × His-tagged HPPD + tyrosine, (3) native HPPD + 4-HPP, and (4) native HPPD + tyrosine. HPPD-like activity was observed only in the two reaction mixtures (1 and 3) supplemented with 4-HPP as a substrate, as evidenced by a new peak corresponding to homogentisate (an intermediate in pyomelanin biosynthesis; Fig. 1) on the HPLC chromatogram (Fig. 4A and C). No tyrosinase-like activity was observed in the reaction mixtures containing tyrosine, even when longer incubation times were applied (Fig. 4B and D). Thus, HPPD activity was observed in vitro but tyrosinase activity was not, strongly indicating that the putative HPPD from *F. kingsejongi* indeed belongs to one of the HPPD enzyme families [31]. Additionally, the kinetic study revealed that the specific activity of *F. kingsejongi* HPPD was 1.2 ± 0.03 μmol homogentisate/min/mg-protein, which was approximately 70 times higher than *Ralstonia pickettii* HPPD, which was 17.1 ± 0.6 nmol homogentisate/min/mg-protein [18]. Notably, phylogenetic analysis based on amino acid sequences showed that the *F. kingsejongi* HPPD was in a distinct clade, separate from the main clade containing HPPDs from other *Flavobacterium* strains (Additional file 1: Fig. S5). To elucidate the structural differences between *F. kingsejongi* HPPD and *Flavobacterium* HPPDs, amino acid sequence homology analysis (Fig. 5A) and homology-based modelling (Fig. 5B) were performed using *F. kingsejongi* HPPD and its four phylogenically close *Flavobacterium* species (*F. endophyticum*, *F. microcysteis*, *F. noncentrifugens*, and *Flavobacterium* sp. BFFFF). As expected, the amino acid sequence homology was relatively high among all five *Flavobacterium* HPPDs. However, homology-based modelling showed that the computed cavity sizes of the active site regions of the five HPPDs were significantly different from the smallest cavity size (883 Å^3^) of *F. kingsejongi* HPPD and the largest cavity size (3844 Å^3^) of *F. microcysteis* HPPD*.* Although the computed cavity size of the active site region cannot be directly related to the activity of HPPDs without biochemical evidence, the spatial difference between the active site regions would affect binding with the substrate 4-HPP, inevitably influencing the kinetics of HPPDs.

Notably, the cavity size of the active site region of *R. pickettii* HPPD was calculated as 2199 Å^3^ (Fig. 5B). It is postulated that this relatively large cavity size might attribute to the reported low specific activity of the *R. pickettii* HPPD in comparison with that of *F. kingsejongi* HPPD, which has a cavity size of 883 Å^3^. Although computational modelling analysis provides feasible explanation for function-structure relationships, additional biochemical studies are required to obtain deeper insight into the binding mechanisms and catalytic performance of HPPDs.

### Bioreactor batch fermentation of *F. kingsejongi*

**Fig. 6:**
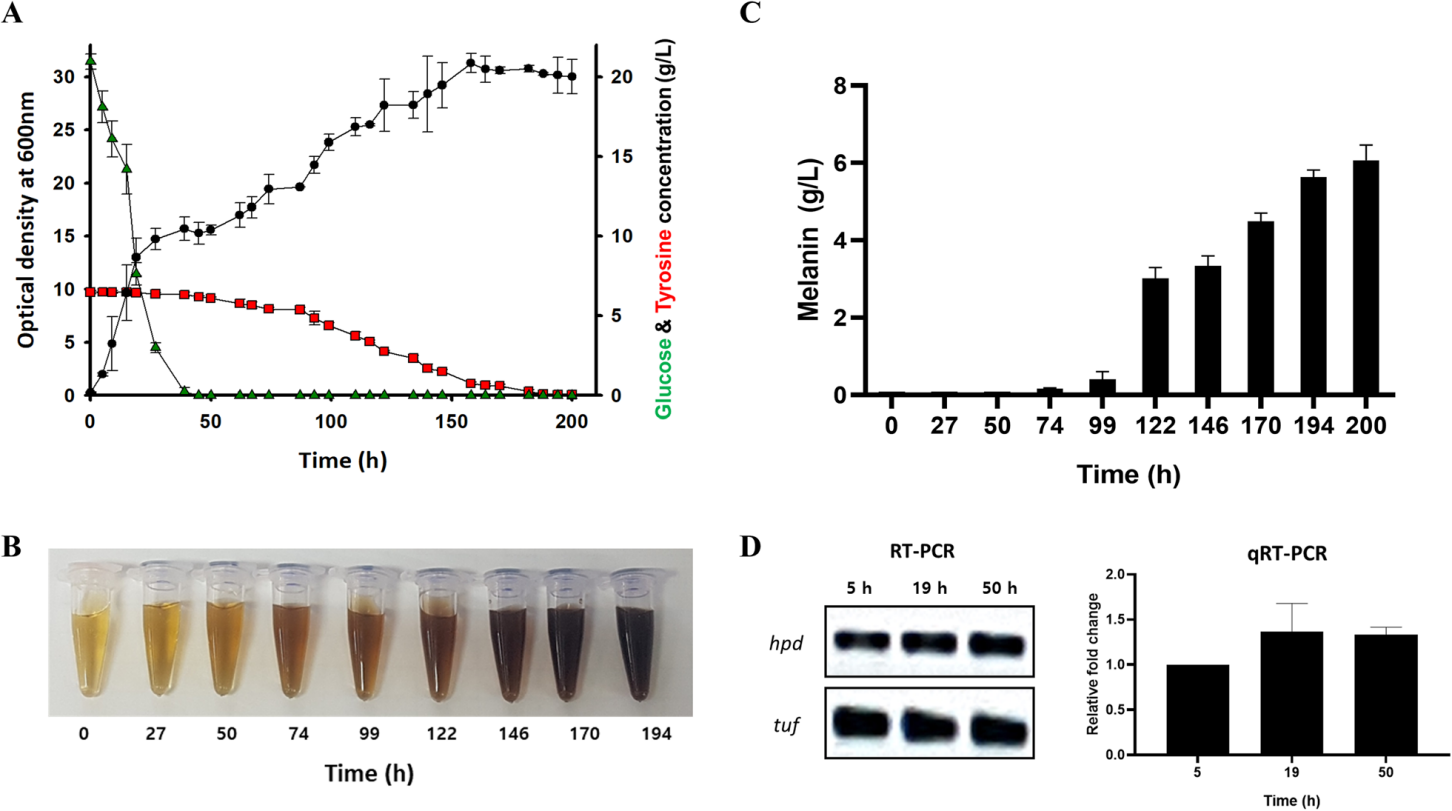
Batch fermentation of melanin-producing *F. kingsejongi* in a 5-L bioreactor. **A** The kinetics of cell growth and the rates of glucose and tyrosine consumption were monitored by measuring optical density at 600 nm (OD_600_) and quantifying the concentrations of glucose and tyrosine present in the culture broth. OD_600_ is represented by filled circles (●), residual glucose concentration by green triangles (▲), and residual tyrosine concentration by red rectangles (■). **B** Supernatant was collected from the culture at set time intervals and visually inspected. **C** The kinetics of *F. kingsejongi* melanin production were monitored by quantifying the melanin present in the culture broth during batch fermentation. **D** RT-PCR (left) and qRT-PCR (right) analysis of the *hpd* gene encoding HPPD in *F. kingsejongi* cells collected at 5, 19, and 50 h of fermentation culture*.* The *tuf* gene was used as a reference

Key parameters to consider in the development of microbial processes for commercializing bacterial melanins include titer, conversion yield, and productivity [17]. The choice between wild-type or natural mutant strains and metabolically engineered strains also needs to be considered [33]. Therefore, we investigated the kinetics of growth and melanin production in *F. kingsejongi* by performing batch fermentation in a 5-L bioreactor containing TB medium supplemented with 20 g/L glucose and 10 g/L tyrosine. *F. kingsejongi* showed a diauxic growth pattern [34] at 40–50 h, reaching an OD_600_ of 30 ± 1.2 at 200 h (Fig. 6A). The glucose in the medium was completely consumed at 40 h, after which tyrosine started to be utilized; tyrosine was completely consumed at 195 h. The color of the culture broth changed significantly after 50 h, which coincided with the start of tyrosine consumption (Fig. 6B). The titer of melanin increased from 0.17 ± 0.02 g/L at 72 h to 6.07 ± 0.32 g/L at 200 h (Fig. 6C). The productivity and conversion yield of melanin were 0.03 g/L melanin per hour and 0.6 ± 0.03 g melanin per gram of tyrosine (~ 60%), respectively. For comparison with other melanogenic bacteria, *S. kathirae* can produce up to 1.76 g/L melanin in a suboptimal medium and up to 13.7 g/L melanin in an optimal medium [25]. Most other melanogenic bacteria, such as *Pseudomonas*, *Streptomyces*, and *Bacillus* strains, can produce from 0.1 to 7.6 g/L melanin in both suboptimal and optimal media [35]. Therefore, although the titer of melanin produced by *F. kingsejongi* was much less than 13.7 g/L, we believe there to be significant potential for enhancing the titer and productivity through understanding and manipulation of melanin biosynthesis regulation and networks based on comparative genomics [36]. Notably, although melanin formation and dark broth coloration were detected after 50–74 h of culture, RT-PCR and RT-qPCR showed that the *hpd* gene encoding HPPD was constitutively transcribed during both the glucose consumption (tests at 5 and 19 h) and early tyrosine consumption (tests at 50 h) phases (Fig. 6D). Therefore, the observed diauxic growth pattern, which led to a reduced conversion yield and titer of melanin, might be avoided by optimizing feeding strategies in fed-batch fermentation of an *F. kingsejongi* strain constitutively expressing HPPD [37, 38]. Furthermore, genetic inactivation of the *hmg* gene encoding homogentisate 1,2-dioxygenase, which further oxidizes homogentisate to fumarate and acetoacetate, might enhance the titer of melanin produced by *F. kingsejongi*. Non-genetic interventions can also be applied to enhance melanin titer; for example, the addition of metals such as Cu, Fe, and Mg to the culture medium could act as a stress simulator to enhance the titer of melanin [35].

### Bioreactor batch fermentation of recombinant *E. coli* expressing HPPD

**Fig. 7:**
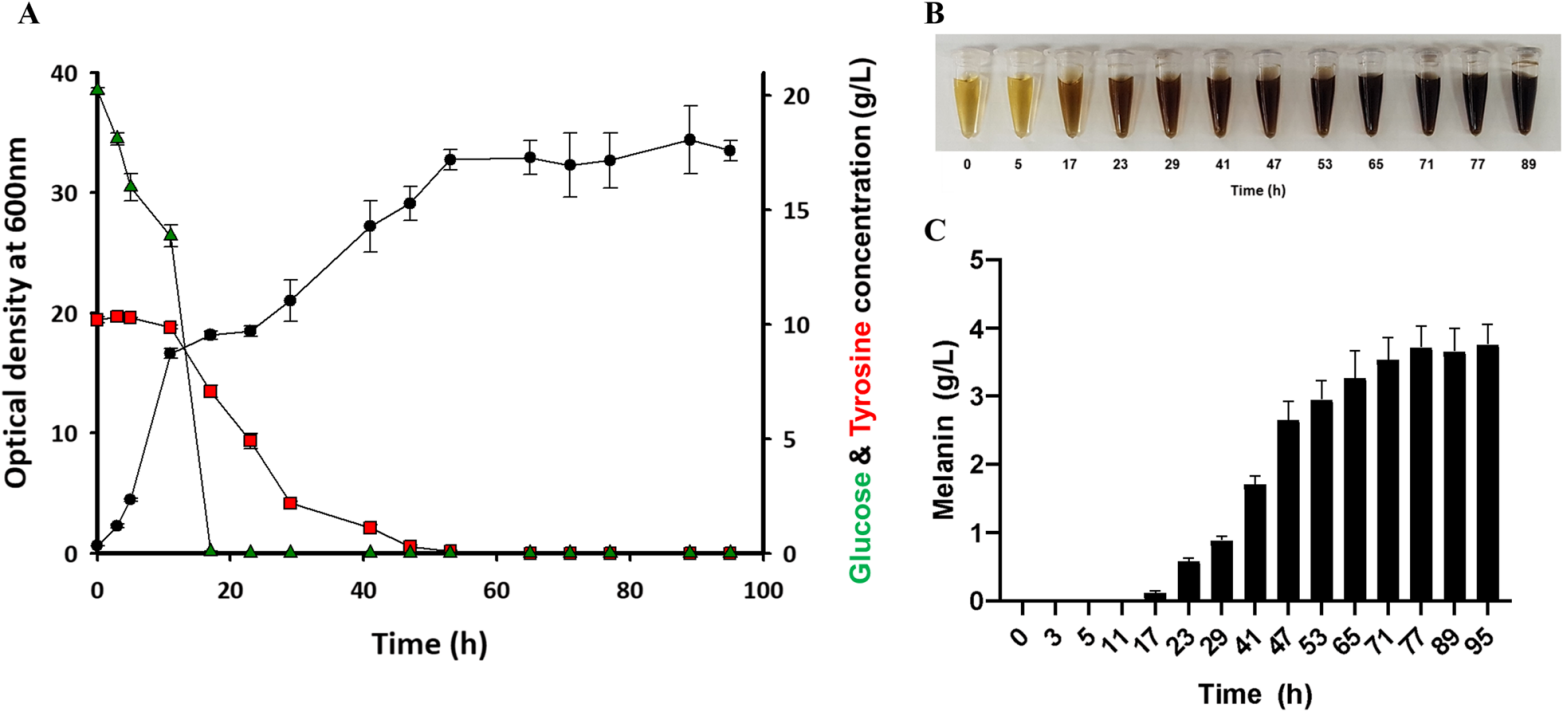
Batch bioreactor fermentation of melanin-producing recombinant *E. coli* expressing the *F. kingsejongi hpd* gene encoding HPPD. **A** The kinetics of cell growth and the rates of glucose and tyrosine consumption were monitored by measuring optical density at 600 nm (OD_600_) and quantifying the concentrations of glucose and tyrosine present in the culture broth. OD_600_ is represented by filled circles (●), residual glucose concentration by green triangles (▲), and residual tyrosine concentration by red rectangles (■). **B** Supernatant was collected from the culture at set time intervals and visually inspected. **C** The kinetics of melanin production by recombinant *E. coli* were monitored by quantifying the melanin present in the culture broth during batch fermentation

Aside from wild-type melanogenic bacteria, recombinant *E. coli* strains have shown potential as melanin producers. In recent studies, recombinant *E. coli* strains expressing HPPD from *P. aeruginosa* [22] and *Ralstonia pickettii* [18] produced melanin titers of 0.21 g/L and 0.31 g/L, respectively. Therefore, we tested the effectiveness of batch fermentation in a bioreactor using a recombinant *E. coli* strain constantly expressing *F. kingsejongi* HPPD, studying the kinetics of its growth and melanin production as with *F. kingsejongi*. Similar to *F. kingsejongi*, the recombinant *E. coli* strain showed a diauxic growth pattern between 11 and 23 h, then reached an OD_600_ of 35 ± 3.2 at 95 h (Fig. 7A). Glucose was completely consumed at 17 h, whereas tyrosine started to be utilized at 11 h and was completely consumed at 41 h. The culture broth color changed significantly at 17 h (Fig. 7B), at which point the melanin titer was 0.12 ± 0.03 g/L; the titer then continuously increased, up to 3.76 ± 0.30 g/L at 95 h (Fig. 7C). Productivity and conversion yield were 0.04 g/L melanin per hour and 0.38 ± 0.03 g melanin per gram of tyrosine (~ 38%), respectively. Although the melanin titer produced by the recombinant *E. coli* was 38% less than that produced by *F. kingsejongi*, the culture time was approximately half that needed for *F. kingsejongi* (95 h vs. 200 h), resulting in higher melanin productivity (0.04 g/L melanin per hour for recombinant *E. coli* vs. 0.03 g/L melanin per hour for *F. kingsejongi*). There is potential for the melanin titer, productivity, and conversion yield of fermentation using recombinant *E. coli* strains to be further increased by optimizing the medium and fermentation method and improving the strains [39]. Altogether, pyomelanin-hyperproducing *F. kingsejongi* strain could serve as a model to elucidate the regulation of the melanin biosynthesis pathway and its networks with other cellular pathways, as well as for understanding the cellular responses of melanin-producing bacteria to environmental changes, including nutrient starvation and other stresses.

## Conclusions

Considering results from the melanin synthesis inhibition test and melanin solubility test, as well as the structural characteristics of purified melanin from both *F. kingsejongi* and *E. coli* expressing *F. kingsejongi* HPPD, we conclude that *F. kingsejongi* produces pyomelanin via the homogentisate pathway and *F. kingsejongi* HPPD exhibited a high specific activity. Finally, large amounts of pyomelanin were obtained by bioreactor batch fermentation using both *F. kingsejongi* and recombinant *E. coli* expressing *F. kingsejongi* HPPD. Both *F. kingsejongi* and recombinant *E. coli* expressing *F. kingsejongi* HPPD showed high fermentation productivity, demonstrating that both strains could be used for the large-scale production of bacterial pyomelanin.

## Methods

### Culture conditions

*Flavobacterium kingsejongi* KCTC 42908^T^ and *S. avermitilis* ATCC 31267^T^ were aerobically grown at 25 °C in flasks containing Luria–Bertani (LB) medium (Gibco, Life Technologies Corporation, Detroit, MI, USA; tryptone 10 g/L, yeast extract 5 g/L, NaCl 5 g/L; shaken at 250 rpm) and on LB agar plates. *E. coli* strain TOP10 was grown at 30 °C in flasks containing LB medium (shaken at 250 rpm) and on LB agar plates containing 100 μg/mL ampicillin (Generay Biotech, Shanghai, China).

### Melanin synthesis inhibition test

To investigate eumelanin synthesis in *F. kingsejongi*, cells were grown at 25 °C for 16 days on LB agar plates supplemented with: (1) 1 g/L tyrosine (Sigma-Aldrich, St. Louis, MO, USA), (2) 100 mg/L kojic acid (Sigma-Aldrich), or (3) 1 g/L tyrosine + 100 mg/L kojic acid. As positive and negative controls, eumelanin-producing *S. avermitilis* 31267^T^ and non-melanin-producing *E. coli* TOP10 were grown on LB plates identical to those used to grow *F. kingsejongi* (25 °C for 7 days for *S. avermitilis*, 37 °C for 6 days for *E. coli*). Similarly, pyomelanin synthesis was investigated by growing *F. kingsejongi,* recombinant *E. coli* expressing HPPD, and *E. coli* on LB agar plates supplemented with: (1) 1 g/L tyrosine, (2) 1 g/L tyrosine + 50 μg/L sulcotrione (Sigma-Aldrich, St. Louis, MO, USA), or (3) 1 g/L tyrosine + 100 μg/L sulcotrione.

### Extraction and analysis of melanin

After flask culture of *F. kingsejongi* and *E. coli* expressing HPPD in LB medium supplemented with 1 g/L tyrosine, cell-free culture medium was collected, adjusted to pH 2.0 by adding 1-N HCl, and kept at 25 °C for 7 days. Next, the culture medium was boiled in a water bath for 1 h and then centrifuged at 10,000×*g* for 15 min. The collected black pellet was washed three times with 0.1-N HCl and soaked in absolute EtOH. The EtOH-soaked melanin pellet was incubated for 10 min in a boiling water bath and then stored at 25 °C for 1 day. Finally, the melanin pellet was washed twice with absolute EtOH, air-dried, and weighed on an electronic scale. Three independent measurements were taken, and their mean was calculated.

### Characterization of melanin extracted from culture broth

A JASCO V-770 ultraviolet–visible (UV–vis) spectrometer (JASCO, Oklahoma City, OK, USA) was used to analyze the spectra from 200 to 1100 nm of melanin pigments purified from *F. kingsejongi*, *S. avermitilis*, and *E. coli*, as well as a commercial eumelanin; samples were measured in a quartz cuvette at room temperature. The Fourier transform infrared (FT-IR) spectra of the four melanins were measured with a Nicolet iS50 FT-IR spectrometer (Thermo Fisher Scientific, Waltham, MA) in attenuated total reflectance (ATR) mode using a diamond crystal. The melanin–KBr disc samples for FT-IR spectrum measurements were prepared by drying the melanin powder in a vacuum oven at 30 °C, followed by mixing with KBr powder. Proton nuclear magnetic resonance (^1^H NMR) spectra were measured using a 600-MHz NMR spectrometer (JEOL, Tokyo, Japan) at room temperature. For ^1^H NMR measurements, 10 mg of each melanin powder was dissolved in 0.75 mL of 0.5-M sodium deuteroxide aqueous solution and sonicated for 15 min.

### Genome mining and heterologous expression of proteins involved in melanin biosynthesis

A standalone basic local alignment and search tool program package (BLAST + v. 2.2.31; http://www.ncbi.nlm.nih.gov/) was utilized to search for genes in the *F. kingsejongi* genome that encoded oxygenases that might be involved in melanin biosynthesis. Putative tyrosinases were explored by running the blastp program with default parameters against the BLAST + local protein database for the *F. kingsejongi* genome (GenBank Accession Number CP020919). The database was searched for amino acid sequences similar to that of a tyrosinase (P55023.2) from *S. lincolnensis.* Sequences of genes encoding HPPD proteins were extracted from a genome annotation file in GFF format, which was generated by running a locally installed version of the NCBI Prokaryotic Genome Annotation Pipeline [40]. Eight candidate genes were found, encoding: (1) tryptophan 2,3-dioxygenase (744 bp), (2) 4-hydroxyphenylpyruvate dioxygenase (1161 bp), (3) homogentisate 1,2-dioxygenase (936 bp), (4) 3-hydroxyanthranilate 3,4-dioxygenase (540 bp), (5) phytanoyl-CoA dioxygenase (546 bp), (6) phytanoyl-CoA dioxygenase (738 bp), 7) a putative dioxygenase (738 bp), and (8) a hypothetical protein (834 bp). The candidate genes were amplified from gDNA by PCR with gene-specific primers, then cloned into a constitutive expression vector plasmid (pUCM), generating eight expression plasmids (Table 1). The eight plasmids were individually transformed into *E. coli*, and the transformed cells were grown at 30 °C for 2 days on LB agar plates supplemented with 1 g/L tyrosine.

**Table 1:** Strains and plasmids used in this study

Strains and plasmids	Relevant properties	Source or references
Strains		
*E. coli* TOP10	F^−^ *mcr*A Δ(*mrr-hsd*RMS*-mcr*BC) φ80*lac*ZΔM15 Δ*lac*X74 *rec*A1 *ara*D139 Δ(*ara-leu*)7697 *gal*U *gal*K *rps*L (StrR) *end*A1 *nup*G	Invitrogen
*E. coli* BL21(DE3)	F^−^ *ompT gal dcm lon hsdSB(r*_*B*_^−^*m*_*B*_^−^*)* λ*(*DE3*[lacI lacUV5-T7p07 ind1 sam7 nin5]) [malB+]*K-12*(*λS*)*	NEB
*Flavobacterium kingsejongi*		KCTC 42908^T^
*Streptomyces avermitilis*		KCTC 9063^T^
Plasmids		
pUCM	Cloning vector modified from pUC19; constitutive *lac* promoter, Ap	Kim et al. 2010
pUCM_TDO	Constitutively expressing putative tryptophan 2,3-dioxygenase from *F. kingsejongi*, Ap	This study
pUCM_HPPD	Constitutively expressing putative 4-hydroxyphenylpyruvate dioxygenase from *F. kingsejongi*, Ap	This study
pUCM_HGD	Constitutively expressing putative homogentisate 1,2-dioxygenase from *F. kingsejongi*, Ap	This study
pUCM_HAAO	Constitutively expressing 3-hydroxyanthranilate 3,4-dioxygenase from *F. kingsejongi*, Ap	This study
pUCM_PHYH	Constitutively expressing putative phytanoyl-CoA dioxygenase from *F. kingsejongi*, Ap	This study
pUCM_PHYH_HypoP	Constitutively expressing putative phytanoyl-CoA dioxygenase, hypothetical protein (dioxygenase) from *F. kingsejongi*, Ap	This study
pUCM_PutaD	Constitutively expressing putative dioxygenase from *F. kingsejongi*, Ap	This study
pUCM_HypoP	Constitutively expressing putative hypothetical protein (dioxygenase) from *F. kingsejongi*, Ap	This study
pET-21a (+)	f1 ori, T7 promoter, C-terminal His-tag sequence, AmpR	Novagen
pET-21a (+)_HPPD	f1 ori, T7 promoter, inducible expression of His6-tagged HPPD, AmpR	This study

### Expression and purification of the *F. kingsejongi *HPPD-encoding gene using *E. coli*

The *hpd* gene that encoded HPPD in *F. kingsejongi* was amplified from genomic DNA by PCR with gene-specific primers (Table 2). The PCR product was then cloned into the inducible vector pET-21a (+) to construct pET-21a (+)_HPPD plasmids expressing 6 × His-tagged HPPD (Table 1). Cells of *E. coli* strain BL21(DE3) harboring pET-21a (+) or pET-21a (+)_HPPD were cultured at 30 °C (shaking at 250 rpm) in 50 mL LB medium supplemented with 100 μg/ml ampicillin. When the recombinant BL21(DE3) culture reached an optical density at 600 nm (OD_600_) of 0.6–0.8, 1 mM isopropyl β-D-1-thiogalactopyranoside **(**IPTG) was added to the culture medium for protein induction, and cells were cultured further for 3 h. Afterward, cells were harvested by centrifugation (3800 rpm, 20 min, 4 °C), washed twice with 50 mM Tris–HCl buffer (150 mM NaCl, pH 8.0), and then resuspended in 20 mL of the same buffer. The resuspended cells were broken up by ultrasonication on ice at 30% power for 5 min (15 pulse cycles of 5 s on and 10 s off), and the supernatant fraction containing the crude soluble proteins was separated from the cell debris by centrifugation (4000 rpm, 4 °C, 30 min). The supernatant was loaded into a GE ÄKTA fast protein liquid chromatography system (GE Healthcare, Chicago, IL, USA) equipped with the HisTrap FF affinity chromatography column (GE Healthcare) and equilibrated with 50 mM Tris–HCl buffer (150 mM NaCl, pH 8.0). Bound proteins were eluted at a flow rate of 1 mL/min with a linear gradient profile between 0 and 250 mM imidazole. The fractions containing 6 × His-tagged HPPD were immediately pooled, desalted with a PD10 desalting column (GE Healthcare), concentrated in an Amicon Ultra-15 centrifugal filter (Millipore, Burlington, MA, USA), and finally buffer-exchanged with 20 mM Tris–HCl buffer (50 mM NaCl, 1 mM dithiothreitol, and 0.1 mM phenylmethylsulfonyl fluoride; pH 8.0). Samples from each step were analyzed by 10% (w/v) SDS-PAGE (sodium dodecyl sulfate polyacrylamide gel electrophoresis) using Coomassie brilliant blue staining. Using the Bradford method, the concentration of the purified 6 × His-tagged HPPD was determined to be 0.4 μg/μL. The purified 6 × His-tagged HPPD was stored in 50% (v/v) glycerol at − 80 °C before use.

**Table 2:** Primers used in this study

Gene	Primer sequence	Enzyme site
Tryptophan 2,3-dioxygenase	F: 5′-gcTCTAGAgcaggaggattacaaaatgtaccatcaggttaatgaa-3′	*Xba*I
	R: 5′-aaggaaaaaaGCGGCCGCttaaacgtttttttcttctccc-3′	*Not*I
4-Hydroxyphenylpyruvate dioxygenase	F: 5′-gcTCTAGAgcaggaggattacaaaatgtcaaaagaagtaaaatcagt-3′	*Xba*I
	R: 5′-aaggaaaaaaGCGGCCGCttataacgttcctcttttttctt-3′	*Not*I
Homogentisate 1,2-dioxygenase	F: 5′-gcTCTAGAgcaggaggattacaaaatgctgaaaggttttgaactaa-3′	*Xba*I
	R: 5′-aaggaaaaaaAGCGGCCGCttactctacccaggacttat-3′	*Not*I
3-Hydroxyanthranilate 3,4-dioxygenase	F: 5′-gcTCTAGAgcaggaggattacaaaatggcaatagcaaaaccgtt-3′	*Xba*I
	R: 5′-aaggaaaaaaGCGGCCGCttatctttttagggcataccg-3′	*Not*I
Phytanoyl-CoA dioxygenase	F: 5′-gcTCTAGAgcaggaggattacaaaatggatcactataaagaacaag-3′	*Xba*I
	R: 5′-aaggaaaaaaGCGGCCGCttagacaacctgtcccc-3′	*Not*I
Phytanoyl-CoA dioxygenase, hypothetical protein	F: 5′-gcTCTAGAgcaggaggattacaaaatggatcactataaagaacaag-3′	*Xba*I
	R: 5′-aaggaaaaaaGCGGCCGCtcagcgttgaaaaacctcc-3′	*Not*I
Putative dioxygenase	F: 5′-gcTCTAGAgcaggaggattacaaactacacgttatgaaaaggaaa-3′	*Xba*I
	R: 5′-aaggaaaaaaGCGGCCGCttatccattcactttaatagtgt-3′	*Not*I
Hypothetical protein	F: 5′-gcTCTAGAgcaggaggattacaaaatggcaacactgcagcc-3′	*Xba*I
	R: 5′-aaggaaaaaaGCGGCCGCttaaccgaattttaccgatgt-3′	*Not*I

Capital letters indicate restriction enzyme sites. Underlines indicate ribosomal binding sequences

### In vitro HPPD activity and kinetics assay

In vitro HPPD activity assays were carried out with purified 6 × His-tagged HPPD or crude protein extract containing HPPD. To determine the activity of HPPD, 8 μg of the purified 6 × His-tagged HPPD (0.4 μg/μL) was added to a 130-μL assay mixture consisting of 100 mM Tris–HCl (pH 7.5), 50 mM ascorbate, and 2 mM 4-hydroxyphenylpyruvate (4-HPP). To prepare the crude protein extract containing HPPD, the IPTG-induced cells were washed, resuspended in 100 mM Tris–HCl (pH 7.5, 50 mM ascorbate), broken up, and then centrifuged. HPPD activity in the crude protein extract was tested by suspending the concentrated extracts in 100 mM Tris–HCl (pH 7.5, 50 mM ascorbate) and adding 2 mM 4-HPP. Similarly, to determine the extent to which HPPD showed tyrosinase-like activity, 8 μg of the purified 6 × His-tagged HPPD (0.4 μg/μL) was added to a 130-μL assay mixture consisting of 100 mM sodium phosphate (pH 7.0), 0.01 mM CuSO_4_, and 3 mM tyrosine. To test the tyrosinase activity of the crude protein extracts, the concentrated extracts were suspended in 100 mM sodium phosphate (pH 7.0) with 0.01 mM CuSO_4_, and 3 mM tyrosine was added. All in vitro assays were performed in a reaction volume of 150 μL at 30 °C and stopped at predetermined time points by adding 20% (w/v) trifluoroacetic acid (TFA). The in vitro assay samples were filtered and analyzed with an Agilent 1200 series HPLC system equipped with a photodiode array detector (Agilent, Santa Clara, CA, USA), using a ZORBAX SB-C18 column (inner diameter, 4.6 mm; length, 250 mm; particle size, 5 μm; Agilent, USA). The mobile phase (70% water, 30% acetonitrile, 0.1% TFA) was made to flow at 1 mL/min for the HPPD activity assay and 0.5 mL/min for the tyrosinase activity assay, and the column temperature was maintained at 30 °C. To determine the specific activity of HPPD, three different concentrations (1.6, 3.2, and 8 μg) of purified 6 × His-tagged HPPD were added to a 150-μL assay mixture consisting of 100 mM Tris–HCl (pH 7.5), 50 mM ascorbate, and 2 mM 4-hydroxyphenylpyruvate (4-HPP). The reaction mixture was incubated for 15 min at 30 °C and immediately quenched by adding 20% (w/v) trifluoroacetic acid (TFA). The homogentisate formation rate was determined by quantification of homogentisate present in the reaction mixture by HPLC, as described above, and the specific activity of HPPD was calculated as μmol homogentisate/min/mg-protein. Commercial standards (4-HPP, homogentisate, L-DOPA, and tyrosine) were purchased from Sigma-Aldrich.

### Analysis of mRNA expression level of HPPD

For the reverse-transcription polymerase chain reaction (RT-PCR) and quantitative reverse-transcription PCR (qRT-PCR) of the *hpd* gene, total RNA was extracted from *F. kingsejongi* cells in the mid-exponential phase using the Hybrid-R™ RNA Purification Kit (GeneAll Biotechnology, Seoul, South Korea). A cDNA library from the total RNA sample was synthesized using the ReverTra™ Ace qPCR RT Kit (Toyobo, Osaka, Japan). The RT-PCR was conducted on a T100™ Thermal Cycler (Bio-Rad, Hercules, CA, USA). The *tuf* gene, encoding the translation elongation factor Tu, was served as a reference gene [41]. qRT-PCR was performed on a Rotor-Gene Q PCR machine (QIAGEN, Hilden, Germany) with SensiFAST™ SYBR^®^ No-ROX one-step kit (Bioline, Cincinnati, OH, USA). Quantification was carried out using the comparative Ct (2^− Δ ΔCT^) method [42]. The primers used for RT- and qRT-PCR were Tuf_forward (5′-ATTCCAACAACTCAGCATCAT C-3′: the *tuf* gene), Tuf_reverse (5′-AGTATGAAACTGCTACCCGTC-3′: the *tuf* gene), Hpd_forward (5′-CTC CAATCAACGAGCACCTTA-3′: the *hpd* gene), and Hpd_reverse (5′- TCTTTGTAGCCC GGAAGAAAC-3′: the *hpd* gene).

### Phylogenetic analysis of HPPD

The phylogenetic position of *F. kingsejongi* HPPD was calculated based on the amino acid sequences of *F. kingsejongi* HPPD and HPPDs from other *Flavobacterium* strains. Evolutionary distances between HPPDs were calculated using the model of Jukes TH and Cantor CR [43], and phylogenetic trees were created using the neighbor-joining method [44], the maximum likelihood method [45, 46], and the unweighted pair group method with arithmetic mean [47]. The resulting trees were subjected to bootstrap analyses [48] based on 1000 resamplings.

### Homology modelling and structural analysis of HPPD

The protein structures of five HPPDs from *F. kingsejongi* and its four phylogenically close *Flavobacterium* species [*F. endophyticum* (GenBank Access Number: NR_145655), *F. microcysteis* (GenBank Access Number: NZ_VFJE00000000), *F. noncentrifugens* (GenBank Access Number: NZ_BKAI00000000), and *Flavobacterium* sp. BFFFF1 (GenBank Accession Number: NKJE010000000)], and the HPPD of *R. pickettii* (GenBank Accession Number: KN050646_JOVL01000000) were computationally predicted using I-TASSER [49]. Molecular docking of the substrate 4-HPP into the model structures and calculation of the cavity sizes were performed using the AutoDock Vina software (v 1.1.2.) [50].

### Batch fermentation of *F. kingsejongi* in a 5-L bioreactor

To evaluate the potential of melanin production by *F. kingsejongi* in a 5-L jar bioreactor, an overnight culture of *F. kingsejongi* was transferred to 100 mL of a modified Terrific Broth (TB) medium (20 g/L glucose, 12 g/L tryptone, 24 g/L yeast extract, 2.31 g/L KH_2_PO_4_, and 12.59 g/L K_2_HPO_4_) in a 500-mL flask, then aerobically grown at 25 °C until the OD_600_ reached 2–3. The preculture was then transferred into a 5-L jar fermenter (BioFlo 320, Eppendorf, Framingham, MA, USA) containing 1.5 L modified TB medium supplemented with 10 g/L tyrosine. Fermentation was carried out at 25 °C with an air flow rate of 1.5 vol/vol/min (vvm). The dissolved oxygen (DO) level was maintained at 30% by supplying pure O_2_ gas or adjusting the agitation rate from 300 to 600 rpm. The pH was maintained at 7.0 by automatic addition of 14% (v/v) NH_4_OH and 2-N HCl.

### Batch fermentation of recombinant *E. coli* expressing HPPD in a 5-L bioreactor

TB medium was used to evaluate the potential of melanin production using recombinant *E. coli* expressing *F. kingsejongi* HPPD in a 5-L jar bioreactor. An overnight culture of recombinant *E. coli* expressing HPPD, grown at 30 °C in a 500-mL flask containing 100 mL of modified TB medium with 100 μg/ml ampicillin, was transferred into a 5-L jar bioreactor containing 1.5 L modified TB medium supplemented with 10 g/L tyrosine and 100 μg/ml ampicillin. Fermentation was carried out at 30 °C with an air flow rate of 1.5 vvm. The DO level was maintained at 30% by supplying pure O_2_ gas or adjusting the agitation rate from 300 to 600 rpm. The pH was maintained at 7.0 by automatic addition of 14% (v/v) NH_4_OH and 2-N HCl.

### Analytical methods

Cell growth was monitored by measuring the OD_600_ of the culture broth using a SpectraMAX Plus 384 spectrophotometer (Bio-Rad, Hercules, CA, USA). Glucose level in the culture medium was quantified with an Agilent 1260 Infinity HPLC system equipped with an Agilent 1200 refractive index detector, using an Aminex HPX-87H column (300 × 7.8 mm; Bio-Rad) with 4 mM H_2_SO_4_ as the mobile phase. The flow rate was 0.7 mL/min, and the column temperature was maintained at 50 °C. To quantify the tyrosine level, the culture medium was diluted tenfold with distilled water, and the pH was adjusted to 9.0 by adding 1-N NaOH, after which the absorbance at 245 nm was read using a SpectraMAX Plus^384^ spectrophotometer.

## Supplementary Information

**Additional file 1: Table S1.** Solubility test of melanin. **Figure S1.** Brown-black pigmentation of *F. kingsejongi*. **A** Colonies of *F. kingsejongi* grown on LB agar plates at 25 ℃. **B** Time-course monitoring of culture broth of *F. kingsejongi* grown in LB medium at 25 ℃. **C** Physiological changes in pigmentation of *F. kingsejongi* grown on LB agar plates supplemented with tyrosine, kojic acid, and tyrosine/kojic acid. Melanin-producing *Streptomyces avermitilis* and unpigmented *E. coli* were used as positive and negative controls, respectively. **Figure S2.** Structural analysis of melanin purified from culture broth containing *E. coli* expressing putative HPPD. **A** UV–vis spectrum, **B** FT-IR spectrum, and **C** ^1^H NMR spectrum of purified melanin from *E. coli* expressing putative *F. kingsejongi* HPPD. **Figure S3.** Inhibition of brown/black pigmentation of *E. coli* expressing HPPD from *F. kingsejongi*. Figure shows the changes in pigmentation of recombinant *E. coli* expressing *F. kingsejongi* HPPD grown on LB agar plates supplemented with either tyrosine or tyrosine + sulcotrione. Melanin-producing *F. kingsejongi* and unpigmented *E. coli* were used as positive and negative controls, respectively. **Figure S4.** Purification of 6 × His-tagged *F. kingsejongi* HPPD, which was overexpressed in recombinant *E. coli*. **A** Chromatogram of bound protein elutions in GE ÄKTA FPLC™ fast protein liquid chromatography (FPLC) system. A red arow below peaks indicates pooled fractions of eluates in FPLC. **B** SDS-PAGE analysis of proteins in purification steps. **Figure S5.** Phylogenetic position of *F. kingsejongi* HPPD among selected *Flavobacterium* HPPDs according to amino acid sequences. The trees were generated using **A** the neighbor-joining method, **B** the maximum likelihood method, and **C** the unweighted pair group method with arithmetic mean. Percentages at the nodes represent the levels of confidence based on bootstrapping with 1000 resamples.

## Data Availability

Additional experimental details and methods (Table S1, Figures S1-S5): Table S1. Solubility test of melanin; Figure S1. Brown-black pigmentation of *F. kingsejongi*; Figure S2. Structural analysis of melanin purified from culture broth containing *E. coli* expressing putative HPPD; Figure S3. Inhibition of brown/black pigmentation of *E. coli* expressing HPPD from *F. kingsejongi*; Figure S4. Purification of 6 × His-tagged *F. kingsejongi* HPPD, which was overexpressed in recombinant *E. coli*; Figure S5. Phylogenetic position of *F. kingsejongi* HPPD among selected *Flavobacterium* HPPDs according to amino acid sequences.
