# Supplementary material for: Melanin biopolymer synthesis using a new melanogenic strain of Flavobacterium kingsejongi and a recombinant strain of Escherichia coli expressing 4-hydroxyphenylpyruvate dioxygenase from F. kingsejongi
Source: Microb Cell Fact. 2022 May 2;21:75. doi: 10.1186/s12934-022-01800-w (PMC9063278; doi:10.1186/s12934-022-01800-w)
Supplement: Supplementary file 1 — Additional file 1: Table S1. Solubility test of melanin. Figure S1. Brown-black pigmentation of F. kingsejongi. A Colonies of F. kingsejongi grown on LB agar plates at 25 ℃. B Time-course monitoring of culture broth of F. kingsejongi grown in LB medium at 25 ℃. C Physiological changes in pigmentation of F. kingsejongi grown on LB agar plates supplemented with tyrosine, kojic acid, and tyrosine/kojic acid. Melanin-producing Streptomyces avermitilis and unpigmented E. coli were used as positive and negative controls, respectively. Figure S2. Structural analysis of melanin purified from culture broth containing E. coli expressing putative HPPD. A UV–vis spectrum, B FT-IR spectrum, and C 1H NMR spectrum of purified melanin from E. coli expressing putative F. kingsejongi HPPD. Figure S3. Inhibition of brown/black pigmentation of E. coli expressing HPPD from F. kingsejongi. Figure shows the changes in pigmentation of recombinant E. coli expressing F. kingsejongi HPPD grown on LB agar plates supplemented with either tyrosine or tyrosine + sulcotrione. Melanin-producing F. kingsejongi and unpigmented E. coli were used as positive and negative controls, respectively. Figure S4. Purification of 6 × His-tagged F. kingsejongi HPPD, which was overexpressed in recombinant E. coli. A Chromatogram of bound protein elutions in GE ÄKTA FPLC™ fast protein liquid chromatography (FPLC) system. A red arow below peaks indicates pooled fractions of eluates in FPLC. B SDS-PAGE analysis of proteins in purification steps. Figure S5. Phylogenetic position of F. kingsejongi HPPD among selected Flavobacterium HPPDs according to amino acid sequences. The trees were generated using A the neighbor-joining method, B the maximum likelihood method, and C the unweighted pair group method with arithmetic mean. Percentages at the nodes represent the levels of confidence based on bootstrapping with 1000 resamples. [file 12934_2022_1800_MOESM1_ESM.docx]

**Additional file 1**

**Table S1.** Solubility test of melanin

| **Test** | **Melanin** | |
| --- | --- | --- |
|  | **Purified melanin**  **(*F. kingsejongi*)** | **Synthetic melanin**  **(Sigma-Aldrich, No. M8631)** |
| Solubility |  |  |
| Water | - | - |
| 1-N NaOH | + | + |
| Ethanol | - | - |
| Acetone | - | - |
| Chloroform | - | - |
| Benzene | - | - |
| Phenol | + | + |
| Precipitation |  |  |
| 1N HCl | + | + |
| 1% FeCl_3_ | + | + |
| Decolorization |  |  |
| 30% H_2_O_2_ | + | + |

**
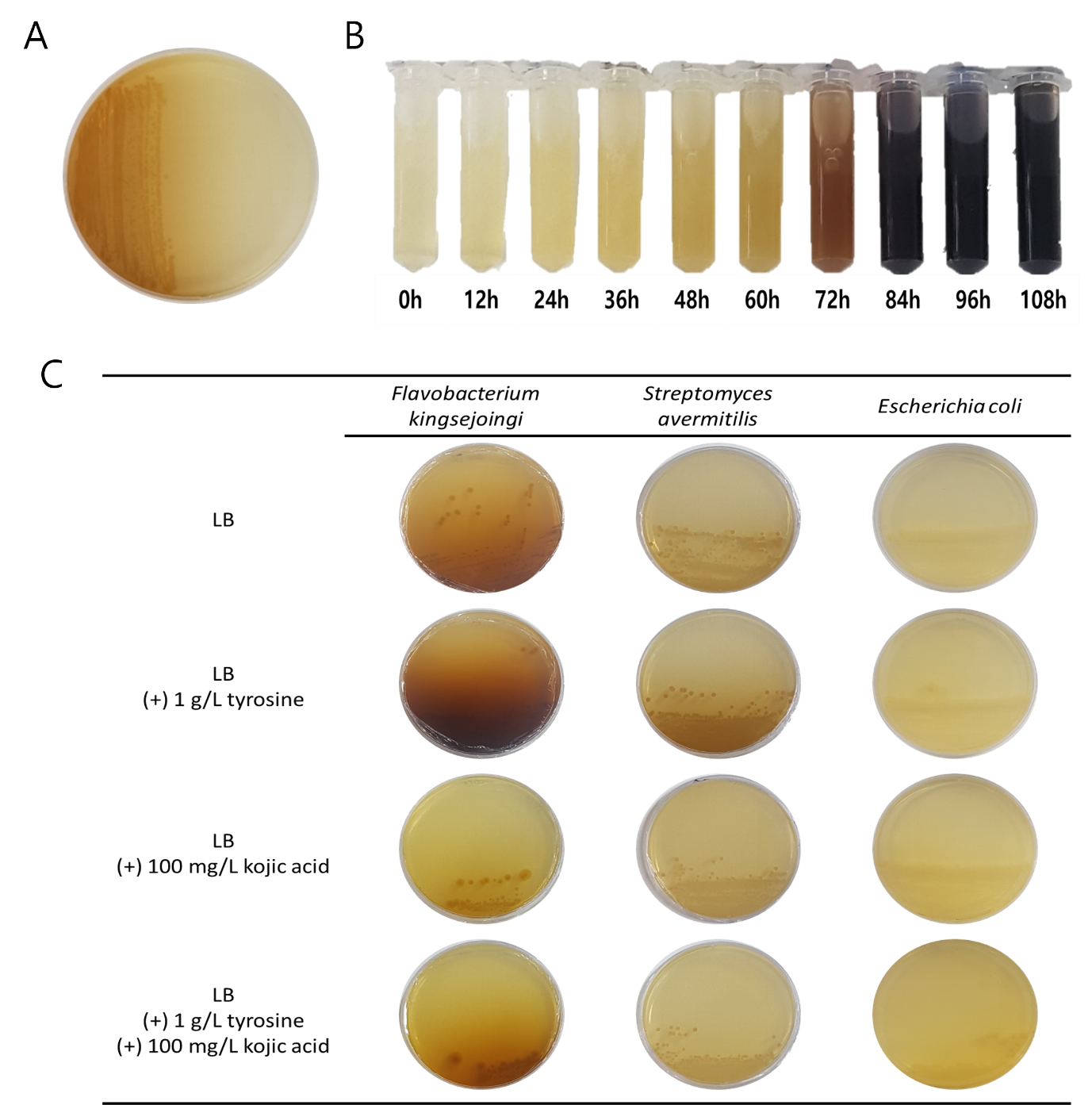
**

**Figure S1.** Brown-black pigmentation of *F. kingsejongi*. (A) Colonies of *F. kingsejongi* grown on LB agar plates at 25 ℃. (B) Time-course monitoring of culture broth of *F. kingsejongi* grown in LB medium at 25 ℃. (C) Physiological changes in pigmentation of *F. kingsejongi* grown on LB agar plates supplemented with tyrosine, kojic acid, and tyrosine/kojic acid. Melanin-producing *Streptomyces avermitilis* and unpigmented *E. coli* were used as positive and negative controls, respectively.

**
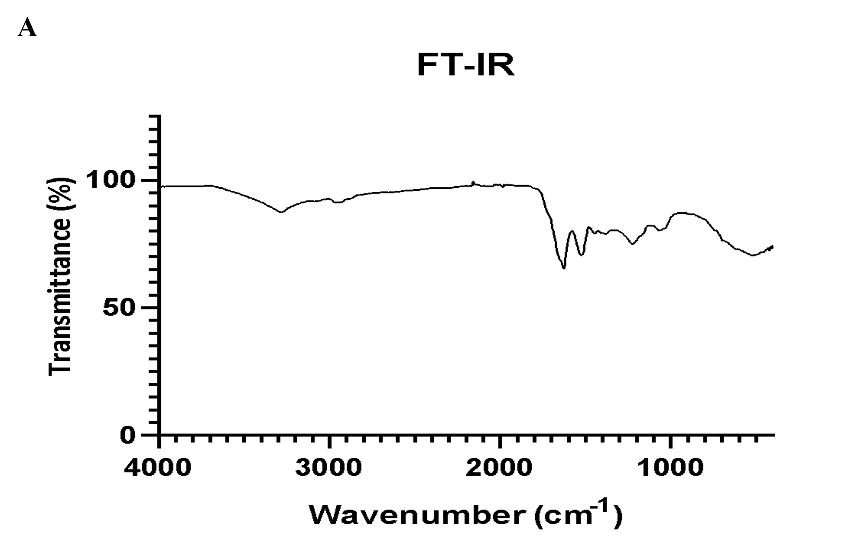

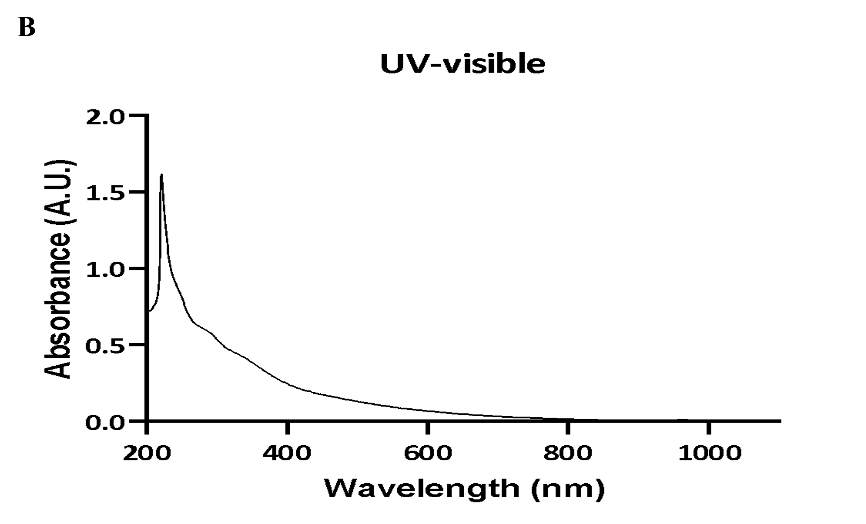

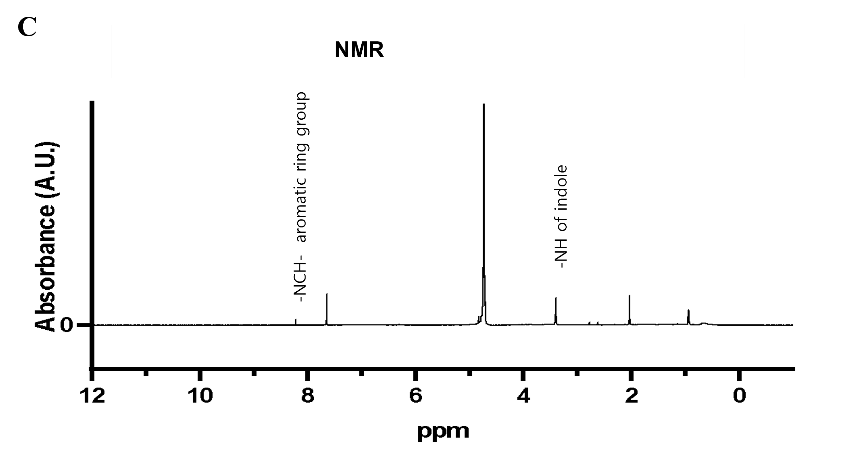
**

**Figure S2.** Structural analysis of melanin purified from culture broth containing *E. coli* expressing putative HPPD. (A) UV-vis spectrum, (B) FT-IR spectrum, and (C) ^1^H NMR spectrum of purified melanin from *E. coli* expressing putative *F. kingsejongi* HPPD.


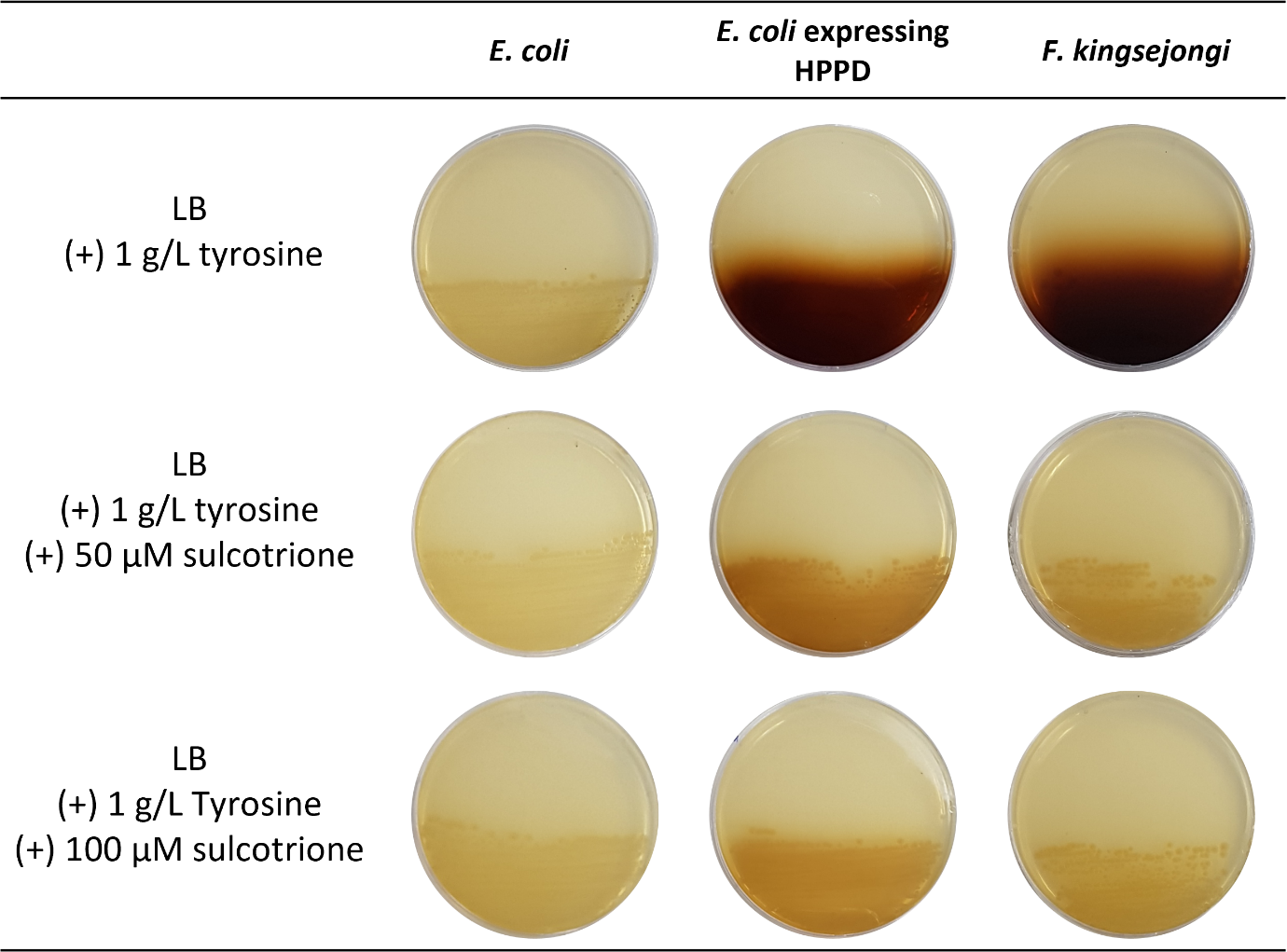


**Figure S3.** Inhibition of brown/black pigmentation of *E. coli* expressing HPPD from *F. kingsejongi*. Figure shows the changes in pigmentation of recombinant *E. coli* expressing *F. kingsejongi* HPPD grown on LB agar plates supplemented with either tyrosine or tyrosine + sulcotrione. Melanin-producing *F. kingsejongi* and unpigmented *E. coli* were used as positive and negative controls, respectively.


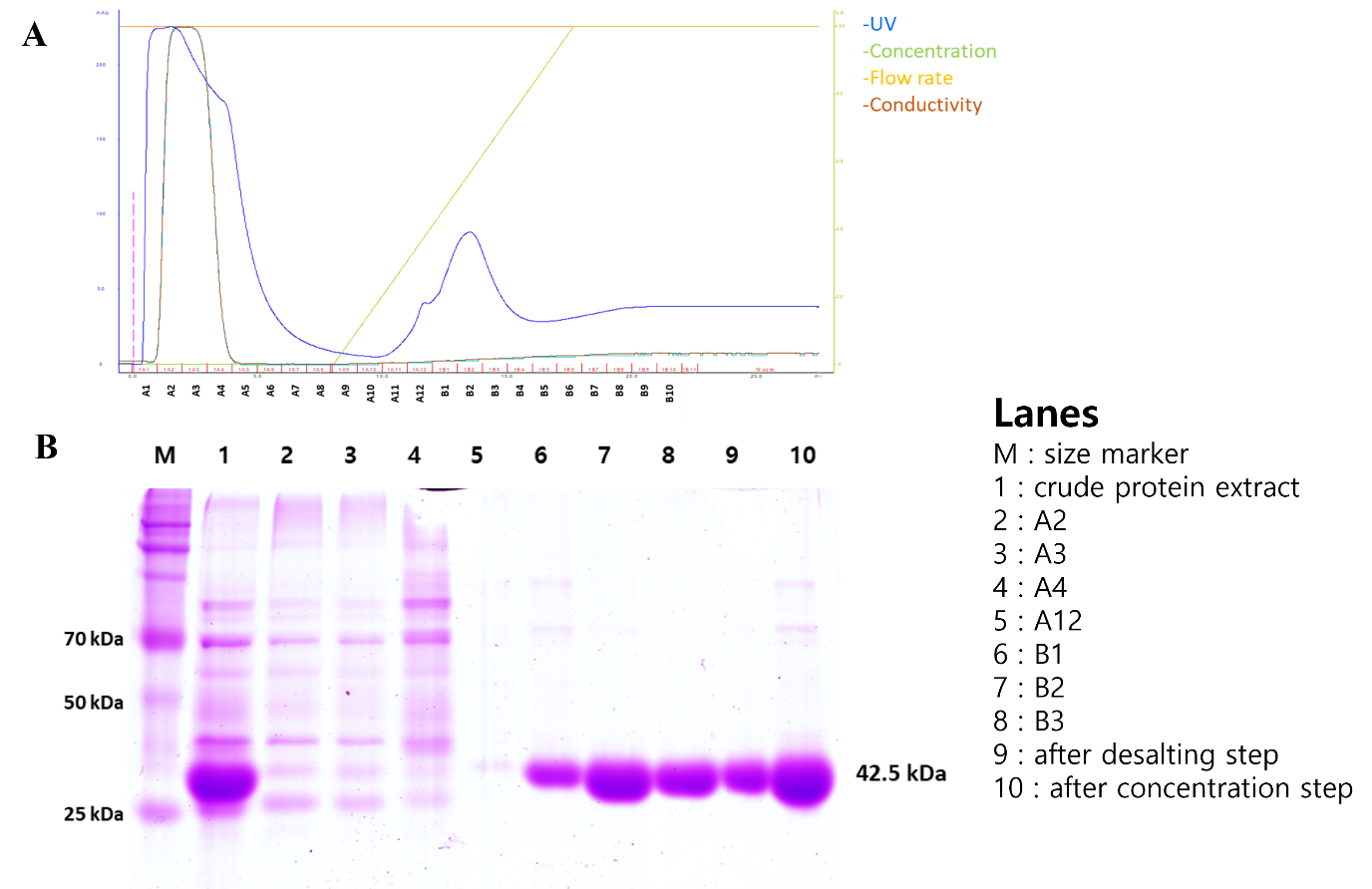


**Figure S4.** Purification of 6×His-tagged *F. kingsejongi* HPPD, which was overexpressed in recombinant *E. coli*. (A) Chromatogram of bound protein elutions in GE ÄKTA FPLC™ fast protein liquid chromatography (FPLC) system. A red arow below peaks indicates pooled fractions of eluates in FPLC. (B) SDS-PAGE analysis of proteins in purification steps.


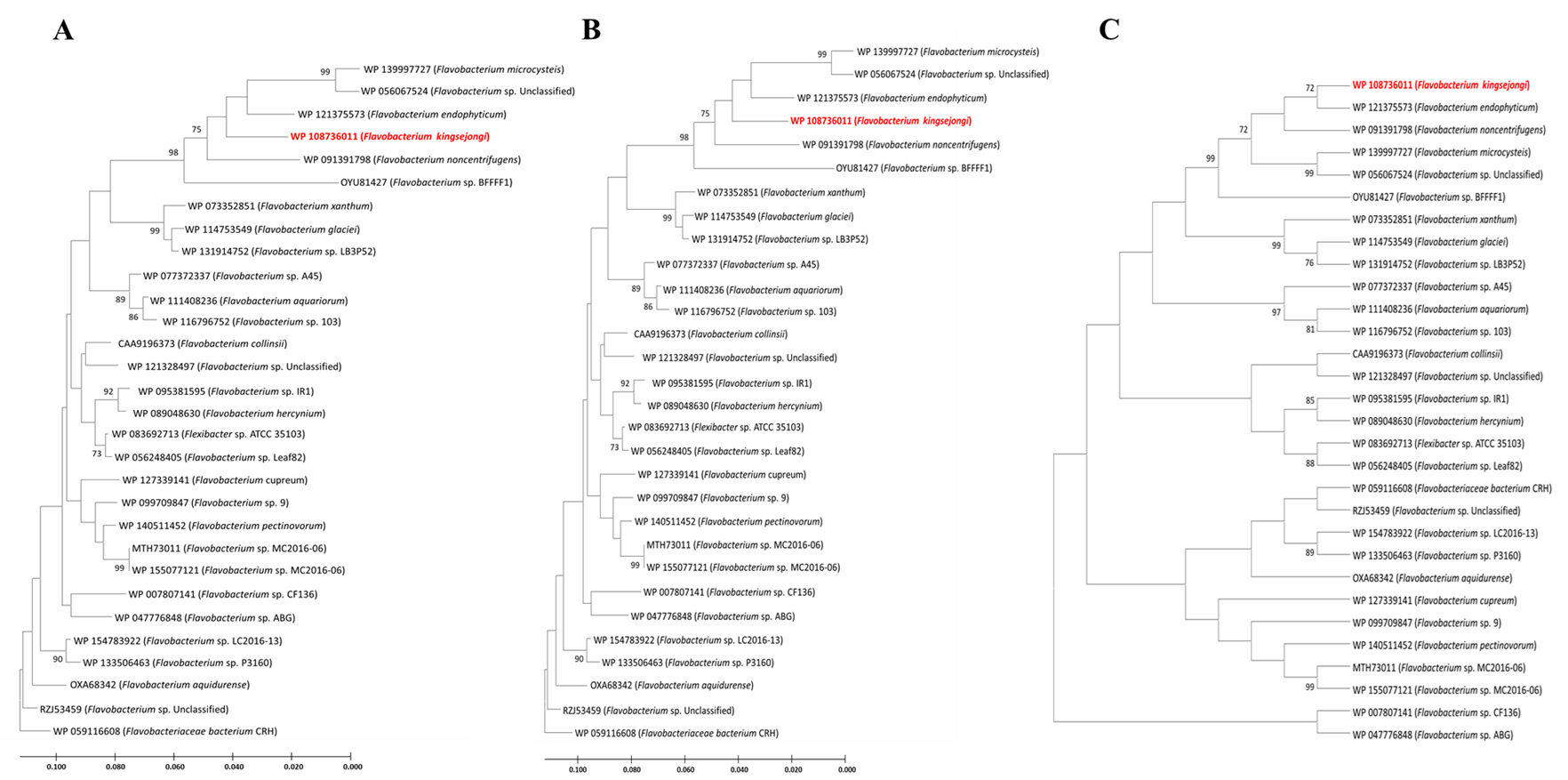


**Figure S5.** Phylogenetic position of *F. kingsejongi* HPPD among selected *Flavobacterium* HPPDs according to amino acid sequences. The trees were generated using (A) the neighbor-joining method, (B) the maximum likelihood method, and (C) the unweighted pair group method with arithmetic mean. Percentages at the nodes represent the levels of confidence based on bootstrapping with 1,000 resamples.
